# Granulocytic sarcoma of the small bowel, greater omentum and peritoneum associated with a CBFβ/MYH11 fusion and inv(16) (p13q22): a case report

**DOI:** 10.1186/1755-7682-4-3

**Published:** 2011-01-21

**Authors:** Paloma Álvarez, Carmen A Navascués, Carlos Ordieres, María Pipa, Iván F Vega, Pablo Granero, José A Alvarez, Manuel Rodríguez

**Affiliations:** 1 Service of Digestive, Hospital Universitario Central de Asturias, Oviedo, Asturias, Spain; 2 Service of Pathology, Hospital Universitario Central de Asturias, Oviedo, Asturias, Spain; 3 Service of Surgery, Hospital Universitario Central de Asturias, Oviedo, Asturias, Spain

## Abstract

**Introduction:**

Granulocytic sarcoma (GS) is an extramedullary disease which is composed of immature myeloid cells or myeloblasts and usually occurs in association with acute myeloid leukemia (AML), as an initial presentation or a relapse. GS has been associated with various cytogenetic abnormalities, particularly with the t(8;21) translocation and less frequently the inv(16) type.

**Case presentation:**

We present a rare case of GS of the small bowel, greater omentum and peritoneum, which caused obstruction, in a patient with AML associated with a CBFβ/MYH11 fusion gene and an inv(16) (p13q22). In this patient there was only mild myeloid hyperplasia in bone marrow aspiration but molecular analysis identified a CBFβ-MYH11 fusion and inv(16) (p13;q22).

**Conclusion:**

Because of its nonspecific clinical and radiologic findings, this entity can be misdiagnosed and can mimic other solid neoplasms, making it a diagnostic challenge. In a GS with no or minimal morphological changes in bone marrow aspiration it is very important to perform a cytogenetic analysis to benefit from the diagnosis and therapeutic strategy.

## Background

Myeloid sarcoma represents an extramedular tumour of myeloblasts and/or immature myeloid cells [1]. Previous terms used to describe this entity include chloroma, extramedullary myeloid tumour, and granulocytic sarcoma (GS). Chloroma was the initial term used to describe these neoplasms, due to gross greenish appearance identified in some lesions [2]. The World Health Organization classification of haematopoietic tumours divides myeloid sarcoma into two major categories [1]. The more common form is GS, composed mainly of myeloblasts, neutrophils, and myeloid precursors. The less common form is monoblastic sarcoma, which is tipically composed of monoblasts and is associated with acute monoblastic leukemia. GS can occur in virtually any anatomic site, with a particular predilection for skin, bone/spine, lymph nodes, soft tissue and genitourinary tract [2,3]. Involvement of gastrointestinal tract is uncommon (7%) and the most frequently involved region in this system is the small bowel (10%) [2,4].

GS may present in association with acute myeloid leukemia (AML), either as an initial presentation or as a relapse. It may also signal impending blast crisis in the setting of a myeloproliferative disorder or leukemic transformation in myelodysplastic syndrome. Less commonly, it may also occur as an isolated mass in non-leukemic patients (primary GS). In this later setting, the majority of untreated patients progress to AML within 11 months [2,5]. GS in the setting of AML occurs most frequently in acute myeloblastic leukemia with maturation (French-American-British [FAB] M2) but has also been described in the other subtypes including FAB M0, M1, M3, M4, M5, and M7 [5].

GS has been associated with various cytogenetic abnormalities, particularly with the t(8;21) translocation [6] and less frequently the inv(16) type [7]. In cases previously reported to involve the small bowel or its mesentery there has been an association with inv(16) or the CBFβ/MYH11 fusion gene or both [7,8].

We present a rare case of GS of the small bowel, greater omentum and peritoneum, which caused obstruction, in a patient with AML type FAB-M2 associated with a CBFβ/MYH11 fusion gene and an underlying inversion of chromosome 16, inv(16) (p13q22).

## Case report

A 41-year-old man was admitted with a 5-day history of intermittent, crampy abdominal pain associated with vomiting and fever. The patient also reported a 2-month history of alternating diarrhea and constipation, abdominal distension and weight loss.

**Figure 1:**
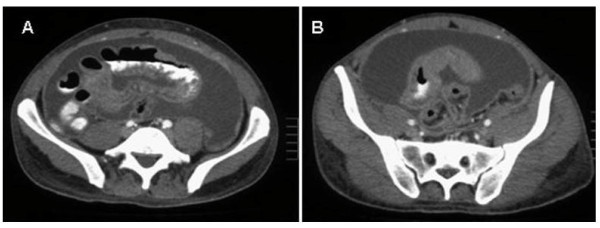
**Abdominal CT scan showing: A) infiltration of the mesentery and the peritoneum and B) ascitis and wall tickening of small bowel**.

**Figure 2:**
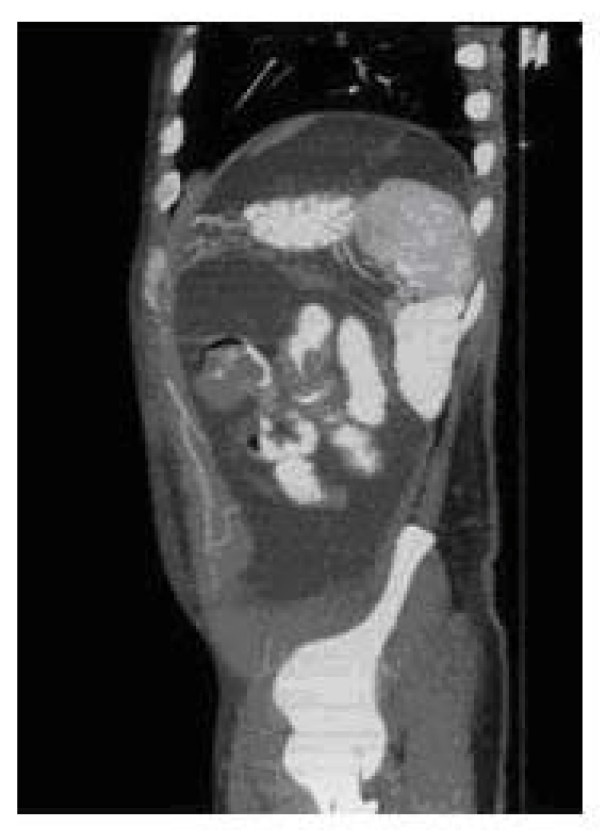
**Abdominal CT scan reveals multiple nodular masses in mesentery and small bowel causing luminal narrowing (sagittal section)**.

**Figure 3:**
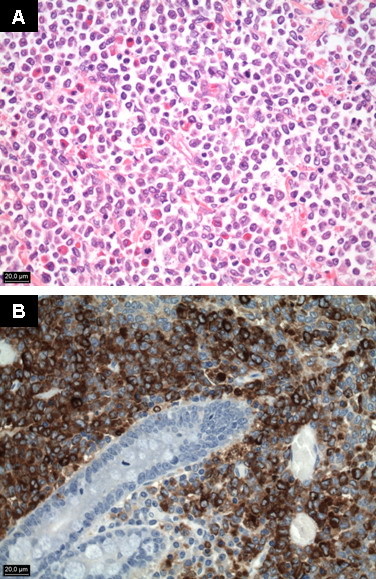
**Photomicrography of small bowel biopsy revealing: A) a infiltrate of poorly differentiated pleomorphic cells with prominent mitotic figures and scant cytoplasm (hematoxylin and eosin stain × 400) and B) Immunohistochemical stain for MPO showing staining of the tumour cells (myeloperoxidase × 400)**.

On admission, the patient was normotensive, afebrile and tachycardic. Generalized abdominal tenderness was noted on palpation, with hyperactive bowel sounds and no guarding or rigidity. The patients also presented bilateral lower extremity edema. No petechia, lymphadenopathy, or hepatosplenomegaly were present. The remainder physical examination was unremarkable. Laboratory examination including a complete blood count, erythrocyte sedimentation rate, liver and renal profile were normal. Stool and blood cultures were negative. Multiple air fluid levels were noted on an upright abdominal radiograph. Enhanced computed tomography (CT) of the abdomen (Figure 1, 2) showed ascitis, dilated loops of small bowel with concentric mural tickening, diffuse peritoneal tickening with multiple peritoneal masses, and infiltration of the mesentery and the greater omentum. No lymphadenopathy was seen. Ultrasound-guided paracentesis was performed, and cytologic evaluation of the ascitic fluid showed a cell population with a blastic appearance presenting multilobular nuclei, prominent nucleoli and cytoplasm scant. Immunohistochemistry study revealed positivity for myeloperoxidase (MPO) and CD68. The neoplastic cells were negative for T-cell markers (CD3, CD5) and for B-cell markers (CD20, CD79a). During hospital stay, the patient's condition steadily deteriorated, and he developed increasing abdominal girth and ascitis and experienced worsening diarrhea, continued weight loss, abdominal pain, and lower extremity edema. With concern for possible myeloid neoplasm and the presence of subacute intestinal obstruction the patient was scheduled to undergo an exploratory laparotomy. On operation, multiple nodular masses in mesentery and small bowel causing luminal narrowing were seen. Laparotomy findings also included ascitis, and multiple masses on the greater omentum and tickening of the peritoneum. Resection of a clear and evident stenotic segment of small bowel with primary anastomosis and biopsies of the greater omentum and the peritoneum was performed. Samples from ascitic fluid were also taken. The jejunal segment that was resected was 14 cm long and had one tumor that was 4 cm long. Histopathology (Figure 3A) showed a transmural infiltrate, extending to the adjoining mesentery, of poorly differentiated pleomorphic cells with numerous interspersed eosinophils. At higher magnification, the cells had prominent mitotic figures, large irregular nuclei, and scant cytoplasm. Immunohistochemical staining (Figure 3B) was strongly positive for MPO, lisozyme and Bc12, and negative for CD10 and Cyclin D1. Similar findings were also found in biopsies of the greater omentum and the peritoneum. Since the final diagnosis was GS, the patient was investigated for leukemia. Flow cytometric analysis performed on the ascitic fluid sample showed the expression of CD13, CD33, CD34, CD117 and HLA-DR. A bone marrow aspiration revealed normal cellularity with preserved megakaryocytes, normoblastic erythroid cells, mild myeloid hyperplasia, no increase in blasts and no dysplasia. Molecular analysis of the bone marrow cells using reverse transcriptase polymerase chain reaction (RT-PCR) identified a CBFβ-MYH11, "type A" fusion transcript with an underlying inversion of chromosome 16, inv(16) (p13;q22). An insertion of FLT3 internal tandem duplication (FLT3-ITD) in exon 14 was found in molecular study of ascitic fluid and intestinal biopsy. According to these data, the diagnoses of acute myeloid leukemia (AML) with inv(16) and FLT3-ITD-positive, M2 in the FAB classification, and granulocytic sarcoma in abdomen were made. Induction chemotherapy for AML with idarubicin and cytosine arabinoside (3+7 regime) was started. The clinical course was complicated by catheter-related sepsis. Abdominal symptoms worsened dramatically in 1 week, so he received only one cycle of treatment. After recovery from post-chemotherapy, radiotherapy was administered. A total dose of 22.4 Gy was administered in 14 fractions over 3 weeks (20 days). After radiotherapy, the patient improved considerably, he gained weight and abdominal pain and distension disappeared. CT scan showed absence of ascitis and a clear decrease in the tickenning of small bowel. Two months after diagnosis of GS, the patient is about to restart chemotherapy.

## Discussion

GS is a rare entity that represents a challenging diagnosis that can present clinically, radiologically, and histopathologically similar to several other processes. This disease has been reported to occur in just about every anatomic location imaginable. GS may present as either isolated or multiple lesions involving one or more organ systems, and lesions may be synchronous or metachronous. In a large series, Yamauchi and Yasuda [5] noted that the majority of reported cases presented as isolated masses. However, patients with multiple lesions, up to four organs systems involved at one time, were also described. In patients with GS involving the small bowel, the age of presentation varies from 8 to 69 years, with the majority occurring in male [4]. Ileum is the most frequently involved region of the gastrointestinal tract [4,9], while its presence in the jejunum is rare [10]. Even more rare is the synchronous involvement of the jejunum and the greater omentum [11], or as in the patient reported here, who had a concomitant involvement of jejunum, greater omentum and peritoneum.

The majority of anatomical sites where GS develops are associated with little to no clinical manifestations and are discovered at autopsy [12]. In contrast, GS in the gastrointestinal tract generally produces symptoms that are often non-specific, as abdominal pain, vomiting, fever, diarrhea, and weight loss [9]. The patients may also present with intussusception [10], gastrointestinal bleeding [13], perforation [9], anasarca and chronic anemia [14], and obstruction [8,11]. Initially, our patient presented with non-specific symptomatology until he was adimitted for obstruction.

Most patients with GS tend to have leukemia at presentation, or they will eventually develop leukemia. The patient reported here is one of the rare cases in which abdominal GS coincided with the onset of AML type FAB-M2 associated with a CBFβ/MYH11 fusion and inv(16) (p13;q22). In a recent review, Zhang et al [15] describe that until 2010, only 20 cases of GS involved with inv(16) have been reported. The chromosome karyotypes of these patients were many and varied. However, all cases had the inv(16) karyotipic abnormality or positive CBFβ/MYH11 fusion gene. AML type FAB-M2 is usually associated with t(8;21) which is more prone to infiltrate extramedullary sites than inv(16). Both of the two types of rearrangements involve the core binding factor gene. The mechanism of the occurrence of GS may be related to the deregulation of the core binding factor transcription factors involved in cell recognition and adhesion [16].

The wide variety of manifestations of GS often make the diagnosis very difficult; gastrointestinal involvement is very unusual and we must maintain a high index of suspicion. The diagnosis of GS can be supported with the use of multiple modalities including histochemical and immunoperoxidase stains, conventional cytogenetics, fluorescent *in situ *hybridization cytogenetics, and flow cytometry. These techniques are especially important in primary GS, in which a previous diagnosis of leukemia or myelodysplastic syndrome has not been made [4,5]. The correct diagnosis can be derived with the aid of histochemical and immunoperoxidase stains such as naphtol-ASD-chloroacetate esterase, lysozime, CD34, CD117, and MPO [2,17], as in our case. Flow cytometry also is helpful in identifying blast populations expressing stem cell (CD34), myeloid (CD13, CD33, CD117, MPO), and/or monocytic (CD11c and CD14) antigens [4]. In our patient, a flow cytometric analysis performed on the ascitic fluid was helpful, as previously reported [14]. Conventional cytogenetics and fluorescent *in situ *hybridization studies are probably most useful in identifying specific characteristics present in previous lesions as a sign of relapse or residual disease. Specific cytogenetic abnormalities most frequently associated with GS include t(8;21) and inv(16) (p13;q22) [4,15]. Most cases of primary GS will develop AML. Although there are reports of primary GS occurring prior the diagnosis of AML [8,11], it is very important to perform bone marrow cytogenetic in a GS before it is coined as primary GS [18]. This fact was demonstrated in the current case report, where the patient showed only mild myeloid hyperplasia in bone marrow aspiration but molecular analysis identified a CBFβ-MYH11 fusion and inv(16) (p13;q22). Hence, it might not be appropriate to label a GS as primary when bone marrow examination shows no or minimal morphological changes but specific cytogenetic abnormalitiy to a particular disease such as CBFβ-MYH11.

GS can be difficult to diagnose, especially in cases that are aleukemic, and it may be mistaken for diffuse large cell lymphoma of the small bowel [7]. Therefore, the diagnostician should maintain a high index of suspicion when encountering an atypical presentation mimicking lymphoma, lymphocytic infiltrate, or autoimmune disease [19]. Timely accurate diagnosis and subclassification is important as prompt treatment with appropriate therapy may prevent the development of a leukemic phase and improve complete response rates and overall survival [7]. In the treatment of GS, systemic chemotherapy, surgical resection, radiotherapy, peripheral stem cell/bone marrow transplantation, or a combination of these approaches are used on a case-by-case basis. However, it remains uncertain what constitutes the best treatment in GS-associated AML patients. Surgery is not usually the first option, and it is only indicated in the event of complications like bleeding or obstruction [10], as in our patient. Although only a few large series comparing treatment modalities of GS are available in the literature [2,20], systemic chemotherapy for AML seems to offer the most benefit. In this regard, Lan et al [21] have pointed out that early diagnosis with biopsy and early chemotherapy seems to improve survival outcome but local radiation or surgery seems to improve symptoms but does not influence survival outcomes.

Leukemia with inv(16) (p13;q22) translocation or CBFβ-MYH11 fusion gene is a especial type which is usually sensitive to chemotherapy and carries a better prognosis than some other leukemia types. It has also been reported that GS reduced the overall survival in t(8;21) AML cases [20] but GS with inv(16) seems not to affect clinical outcome [15]. Further investigation is still necessary to identify the clinical significance of inv(16) of GS and to choose appropriate therapeutic strategy. Activating FLT3 mutations occur in approximately 40% of cytogenetically normal AML patients and the most frequent activating mutations is FLT3-ITD, whose presence is associated with resistence to chemotherapy and inferior outcome [22]. This unfavorable prognostic factor was found in our patient.

## Conclusion

We describe a rare case of abdominal GS in a patient with AML associated with a CBFβ/MYH11 fusion and inv(16) (p13q22). Because of its nonspecific clinical and radiologic findings, this entity can be misdiagnosed and can mimic other solid neoplasms, making it a diagnostic challenge. In a GS with no or minimal morphological changes in bone marrow aspiration it is very important to perform a cytogenetic analysis. Although all patients should be treated with chemotherapy for AML, radiotherapy and surgery are indicated in appropriate clinical settings.

## Consent

Written informed consent was obtained from the patient for publication of this case report and accompanying images. A copy of the written consent is available for review by the Editor-in-Chief of this journal.

## Competing interests

The authors declare that they have no competing interests.

## Authors' contributions

PA, CAN, CO, MP, PG, JAA, MR were involved in the direct care of this patient. In addition, PA and JAA selected the case and were responsible for drafting the manuscript. IFV performed the histopathology on small bowel, greater omentum and peritoneum biopsies. All authors have read and approved the final manuscript.
